# TheCellMap.org: A Web-Accessible Database for Visualizing and Mining the Global Yeast Genetic Interaction Network

**DOI:** 10.1534/g3.117.040220

**Published:** 2017-03-20

**Authors:** Matej Usaj, Yizhao Tan, Wen Wang, Benjamin VanderSluis, Albert Zou, Chad L. Myers, Michael Costanzo, Brenda Andrews, Charles Boone

**Affiliations:** * The Donnelly Centre, University of Toronto, Ontario M5S 3E1, Canada; † Department of Computer Science and Engineering, University of Minnesota-Twin Cities, Minneapolis, Minnesota 55455; ‡ Simons Center for Data Analysis, Simons Foundation, New York, New York 10010; § Lewis-Sigler Institute for Integrative Genomics, Princeton University, New Jersey 08544; ** Department of Molecular Genetics, University of Toronto, Ontario M5S 3E1

**Keywords:** genetic interactions, genetic network, yeast genetics, synthetic genetic array SGA

## Abstract

Providing access to quantitative genomic data is key to ensure large-scale data validation and promote new discoveries. TheCellMap.org serves as a central repository for storing and analyzing quantitative genetic interaction data produced by genome-scale Synthetic Genetic Array (SGA) experiments with the budding yeast *Saccharomyces cerevisiae*. In particular, TheCellMap.org allows users to easily access, visualize, explore, and functionally annotate genetic interactions, or to extract and reorganize subnetworks, using data-driven network layouts in an intuitive and interactive manner.

The majority of the ∼6000 yeast genes are individually dispensable under standard growth conditions, with only a relatively small subset (∼20%) of genes required for viability (Winzeler *et al.* 1999; giaever *et al.* 2002). This large fraction of nonessential genes likely reflects the evolution of extensive buffering against genetic and environmental perturbations (Hartman *et al.* 2001). Genome-scale screens for genetic interactions provide a means to explore this buffering capacity and map a functional wiring diagram of a cell (Costanzo *et al.* 2010, 2016; Vizeacoumar *et al.* 2013). In addition to functional information about genes and their biological pathways, genetic interaction networks may also provide fundamental insights into the genetic architecture underlying the genotype-to-phenotype relationship (Zuk *et al.* 2012; Bloom *et al.* 2013, 2015).

SGA analysis is an automated form of yeast genetics that combines arrays of either nonessential gene deletion mutants, or conditional alleles of essential genes, through a series of robotic manipulations to enable high throughput construction of haploid yeast double mutants and quantitative analysis of genetic interactions (Tong *et al.* 2001; Baryshnikova *et al.* 2010). Genetic interactions identified through SGA analysis can be grouped into two general categories, negative and positive (Costanzo *et al.* 2011). Negative genetic interactions describe double mutants that exhibit a more severe phenotype than expected based on the phenotypes of the corresponding single mutants (Mani *et al.* 2008). Synthetic lethality is an extreme example of a negative interaction where two mutations, each causing little fitness defect on their own, result in an inviable or cell death phenotype when combined as double mutants (Novick and Botstein 1985). Negative interactions are of particular interest because they tend to connect functionally related genes that impinge on the same essential biological process (Costanzo *et al.* 2010, 2016). Conversely, positive genetic interactions describe double mutants exhibiting a less severe phenotype than expected based on the product of the two single mutant phenotypes (Mani *et al.* 2008). Genes encoding members of the same nonessential protein complex are often connected by positive genetic interactions because once the function of the complex is compromised by mutation of the first component, the phenotype cannot be made worse by removal of additional components (Costanzo *et al.* 2011). However, the vast majority of positive interactions do not connect genes within the same nonessential pathway or even functionally related genes, but instead tend to map more general regulatory connections associated with defects in cell cycle progression or cellular proteostasis (Costanzo *et al.* 2016). Despite their mechanistic differences, both negative and positive genetic interactions tend to be highly organized and genes belonging to the same protein complex or pathway often share a similar pattern of genetic interactions (Costanzo *et al.* 2011). Moreover, the set of negative and positive genetic interactions for a given gene, termed the genetic interaction profile of a gene, provides a quantitative measure of gene function, and subsets of genes with highly similar genetic interaction profiles often belong to the same biological process, pathway, and/or protein complex (Costanzo *et al.* 2010, 2016).

In a recent study, we described the construction of a global genetic interaction network for *Saccharomyces cerevisiae* that consists of nearly one million genetic interactions (Costanzo *et al.* 2016). This study not only identified general properties of negative and positive interactions, but it also showed that the global network of genetic interaction profile similarities groups genes into a hierarchical model of a cellular function. For example, at the highest resolution of the profile similarity network, genes are grouped into modules corresponding to protein complexes and pathways. At an intermediate level of network resolution, these groups combine together to define specific biological processes. Finally, at the most general level of network resolution, gene clusters representing biological processes are grouped together into larger modules corresponding to specific cellular compartments (Costanzo *et al.* 2016). This biologically-intuitive organization of genes provides a powerful resource for predicting the function of previously uncharacterized genes. Here, we describe TheCellMap.org, a web-accessible database and visualization tool, designed to facilitate data access and navigation of the global yeast genetic interaction network.

## Materials and Methods

### Genetic interaction screens and analysis

SGA genetic interaction screens and analyses were conducted as described elsewhere (Baryshnikova *et al.* 2010; Kuzmin *et al.* 2014; Costanzo *et al.* 2016).

### Database development

TheCellMap.org is a Rich Internet Application (RIA) that enables efficient querying of large interaction datasets. The back-end of TheCellMap.org is written in Python using the Django web framework connected to a PostgresSQL object-relational database for storage and querying. The web interface is developed using a combination of HyperText Markup Language (HTML5), Cascading Style Sheets (CSS3) and JavaScript for an interactive user experience. An nginx web server links the client and server sides by serving static files and passing Hypertext Transfer Protocol (HTTP) requests through a Web Server Gateway Interface (uWSGI). All labor-intensive requests are processed in python using numpy for fast numerical analysis of the queried data which further decreases the load on a user’s browser.

### Analysis of neighboring gene effect

If mutation of a particular gene results in disruption of an adjacent gene, then the two genes should share many genetic interactions in common and exhibit similar genetic interaction profiles. To explore the impact of neighboring gene effects on genetic interaction profile similarity, we computed the physical distance separating all unique pairs of genes located on the same chromosome. Nonoverlapping gene pairs were then assigned to 100 bp interval bins based on the chromosome distance separating them (*e.g.*, 0–100 bp, …, 4900–5000 bp). Gene pairs with overlapping coding sequences were assigned to 500 bp bins (*e.g.*, −2000 to −1500 bp, …, −500 to 0 bp). The mean and SD of genetic interaction profile similarity was then measured for all gene pairs in the same chromosome distance bin. Finally, the mean profile similarity of all gene pairs within the same chromosome distance bin was compared to the average profile similarity of previously defined “cocomplex” gene pairs (Costanzo *et al.* 2016).

### Data availability

The authors state that all data necessary for confirming the conclusions presented in the article are represented fully within the article.

## Results and Discussion

### Data acquisition, processing, and analysis

A genetic interaction between two genes can be quantified based on measurement and comparison of the single mutant phenotypes, an estimate of the expected double mutant phenotype, and the observed double mutant phenotype. In the case of yeast fitness, the expected double mutant phenotype is typically modeled as a multiplicative combination of single mutant phenotypes, and genetic interactions are measured by the extent to which double mutants deviate from this multiplicative expectation (Phillips *et al.* 2000; Mani *et al.* 2008). In a typical SGA screen, a haploid *MAT*α query mutant strain is crossed to an input array of haploid *MAT***a** yeast mutants to generate an array of heterozygous diploid double mutants. Ultimately, an output array of haploid double mutants is generated through mating, meiosis, and the subsequent selection of haploid double mutant *MAT***a** meiotic progeny (Kuzmin *et al.* 2014). Computational methods have been developed to correct sources of systematic variability associated with high-density yeast colony arrays, providing accurate haploid single and double mutant colony size measurements that serve as a proxy for cell fitness and the basis for quantitative genetic interaction analysis (Baryshnikova *et al.* 2010).

**Figure 1:**
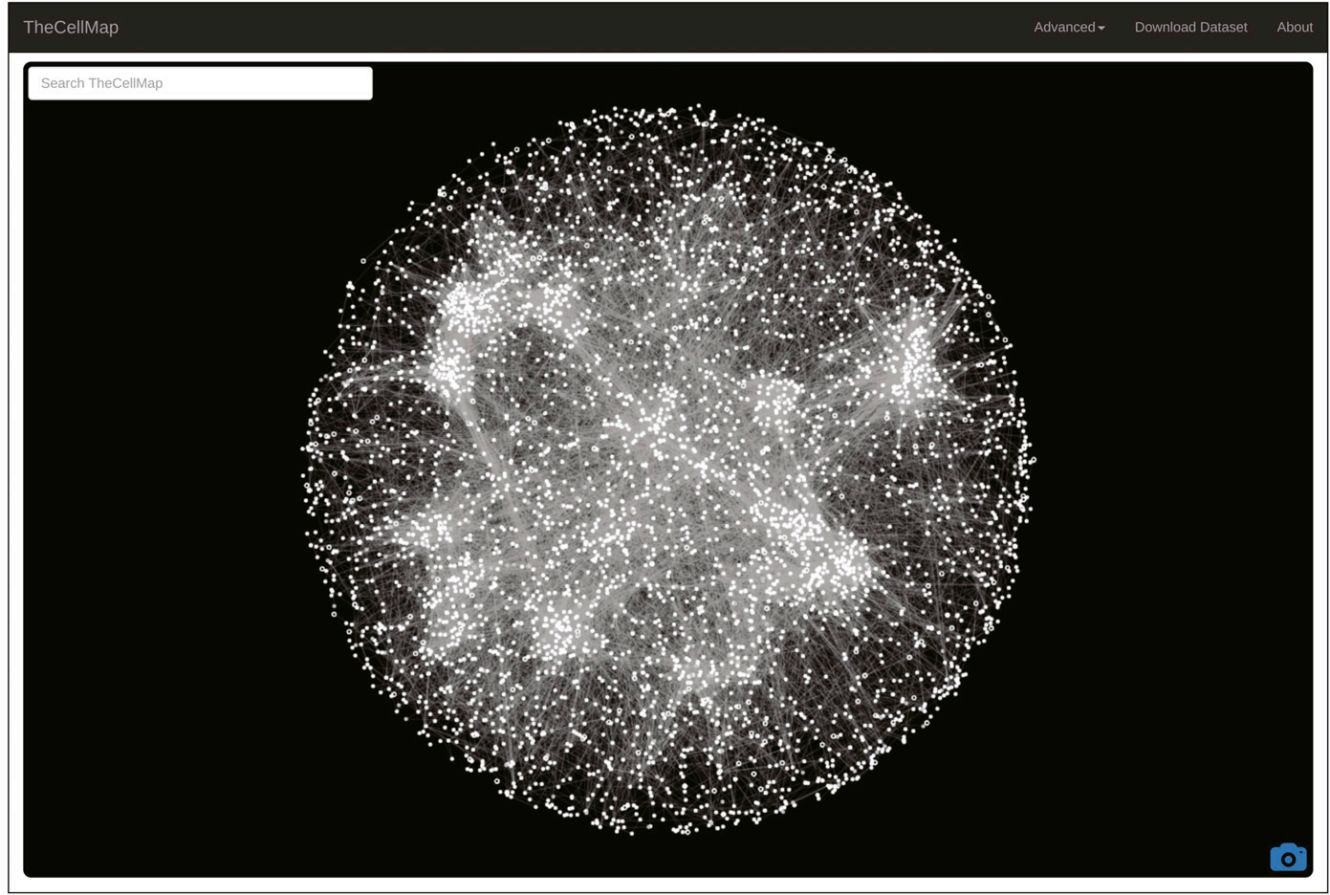
A global genetic interaction profile similarity network. A screenshot of TheCellMap.org home page, which displays the global genetic interaction profile similarity network.

Combining SGA and an automated colony scoring method enabled the construction and analysis of ∼23 million yeast double mutant strains (Costanzo *et al.* 2016). The global genetic interaction network derived from this analysis includes ∼550,000 negative and ∼350,000 positive genetic interactions, and represents ∼90% of all yeast genes as either array and/or query mutants (Costanzo *et al.* 2016). Genetic interaction profile similarities were also measured by computing Pearson correlation coefficients (PCC) between all pairs of yeast mutant strains screened in this study (Costanzo *et al.* 2016). The complete unfiltered dataset, including negative and positive interactions as well as genetic interaction profile similarities, is available for download in various formats from the menu bar on TheCellMap.org home page (Figure 1, “Download Dataset”). In addition to providing access to the dataset, TheCellMap.org also allows users to explore and visualize genetic interaction data in two different formats: (1) a global network based on genetic interaction profile similarities and; (2) networks comprised directly of negative and positive genetic interactions for mutant alleles of selected genes. The mutant alleles largely consist of deletion alleles of nonessential genes and temperature-sensitive (TS) alleles of essential genes, a number of which have multiple different alleles.

### Navigating the global genetic interaction profile similarity network

TheCellMap.org home page shows an interactive version of the global genetic interaction profile similarity network (Figure 1). Nodes represent deletion alleles of nonessential genes or TS alleles of essential genes. Alleles sharing similar genetic interaction profiles that exceed a defined PCC threshold (PCC ≥ 0.2) are connected by an edge in the network. To visualize the network, an edge-weighted, spring-embedded layout algorithm was applied to determine the position of all nodes, such that tightly connected nodes (*i.e.*, genes sharing similar patterns of genetic interactions) are placed proximal to each other whereas less connected nodes are placed farther apart in two-dimensional space (Kobourov 2012). The current genetic interaction profile similarity network consists of 4909 nodes representing 4418 unique genes connected by 34,468 edges. Densely-connected clusters on the global network are enriched for subsets of functionally related genes that represent distinct biological processes and, within each bioprocess, more refined subsets of genes cluster into pathways and complexes (Costanzo *et al.* 2016). To identify gene and allele names, a user can zoom into specific network regions by scrolling on a particular network region or by hovering over individual nodes. Double clicking on a specific node directs a user to the *Saccharomyces* Genome Database (www.yeastgenome.org) Gene Summary Page for the selected gene. The camera icon, located in the bottom right corner of the home page, allows a user to save the network shown on the screen as a Scalable Vector Graphics (SVG) image (Figure 1).

**Figure 2:**
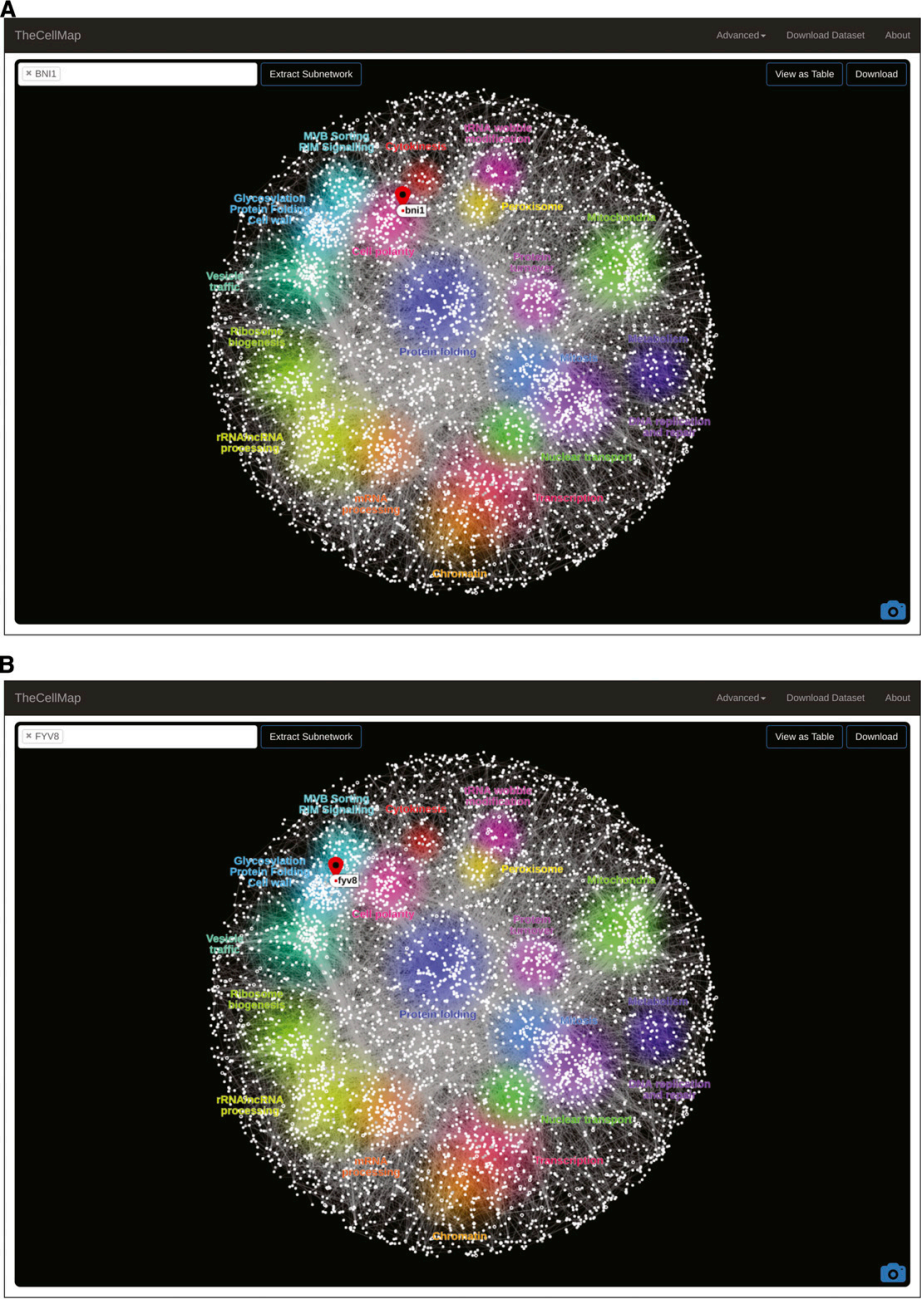
Mapping genes on the genetic interaction profile similarity network. Following a gene search, the position of the gene of interest is shown with a teardrop icon (red). The biological processes represented by each network cluster are also highlighted (colored regions). (A) A gene search revealed the position of *BNI1* on the global genetic interaction network. (B) A gene search revealed the position of a previously uncharacterized gene, *FYV8*, on the global genetic interaction network.

The global genetic profile similarity network provides a powerful resource for predicting gene function because the network position of a given gene can be indicative of its general function (Costanzo *et al.* 2016). Indeed, by exploring gene position on the network systematically, we predicted and validated functions for several poorly characterized genes including essential genes whose functions were not previously appreciated (Costanzo *et al.* 2016). The specific location of a gene(s) can be mapped onto the global profile similarity network by inputting the systematic or common gene name(s) into the search window (Figure 1, upper left) or by selecting a region of the network. Following a gene search or selection of a network region, the position of a selected gene on the network is shown by the appearance of a teardrop icon (Figure 2). Following a gene search, the general biological processes associated with each cluster, as determined by Spatial Analysis of Functional Enrichment (SAFE) (Baryshnikova 2016), are also highlighted with different colors (Figure 2). Thus, proximity to an enriched network cluster should provide insight into the cellular function of a particular gene. For example, *BNI1*, a formin protein important for the nucleation of actin cables that control polarized secretion (Evangelista *et al.* 2003), localizes to the “Cell Polarity” cluster (Figure 2A). Interestingly, a previously uncharacterized gene, *FYV8*, is located within a cluster enriched for genes involved in glycosylation, protein folding, and cell wall biosynthesis, suggesting that this gene may share a similar role with other genes involved in these functions (Figure 2B). A new gene search can be initiated by selecting the “TheCellMap” icon in the menu bar which resets the network (Figure 1 and Figure 2).

**Figure 3:**
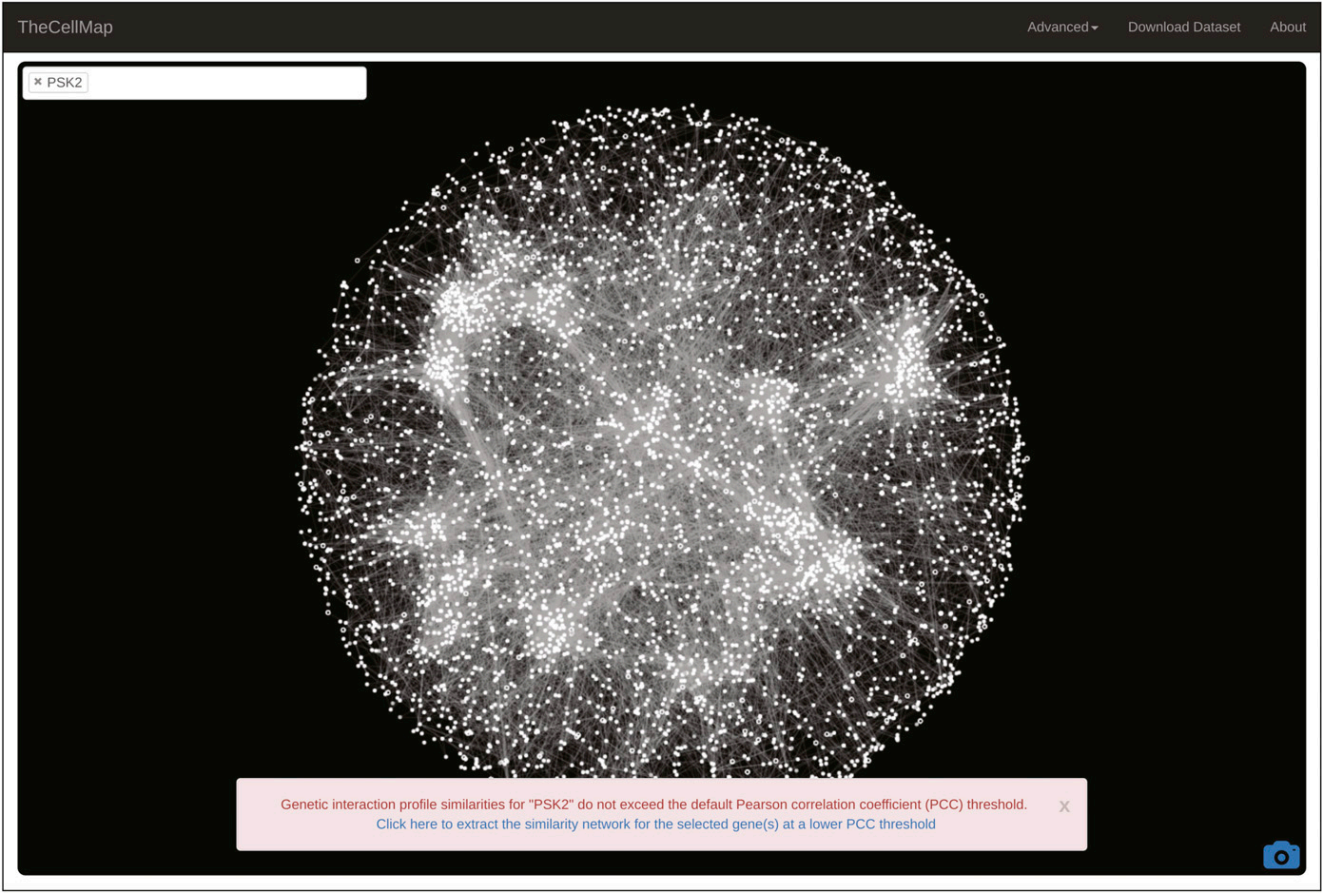
Generating profile similarity networks for genes that do not appear on the global network. A warning message indicates that a gene(s) of interest is not represented on the global network and a link is provided to view a subnetwork of genes that show the strongest profile similarity to the gene(s) of interest. PCC, Pearson correlation coefficient.

As described above, a threshold was applied on profile similarity in order to minimize network complexity and facilitate visualization of the biological process-enriched network clusters (Costanzo *et al.* 2010, 2016). As a result, a gene with a genetic interaction profile that does not meet a minimum similarity (PCC ≥ 0.2) with the genetic interaction profile of any other gene will not appear on the global network. A search for such genes results in a message indicating that the gene of interest is not represented on the global network. Although this gene cannot be visualized on the global network, a subnetwork illustrating the most similar genetic interaction profiles to the gene of interest can be generated by clicking on the hyperlink shown in the message box (Figure 3).

### Extracting gene-specific genetic interaction profile similarity networks

**Figure 4:**
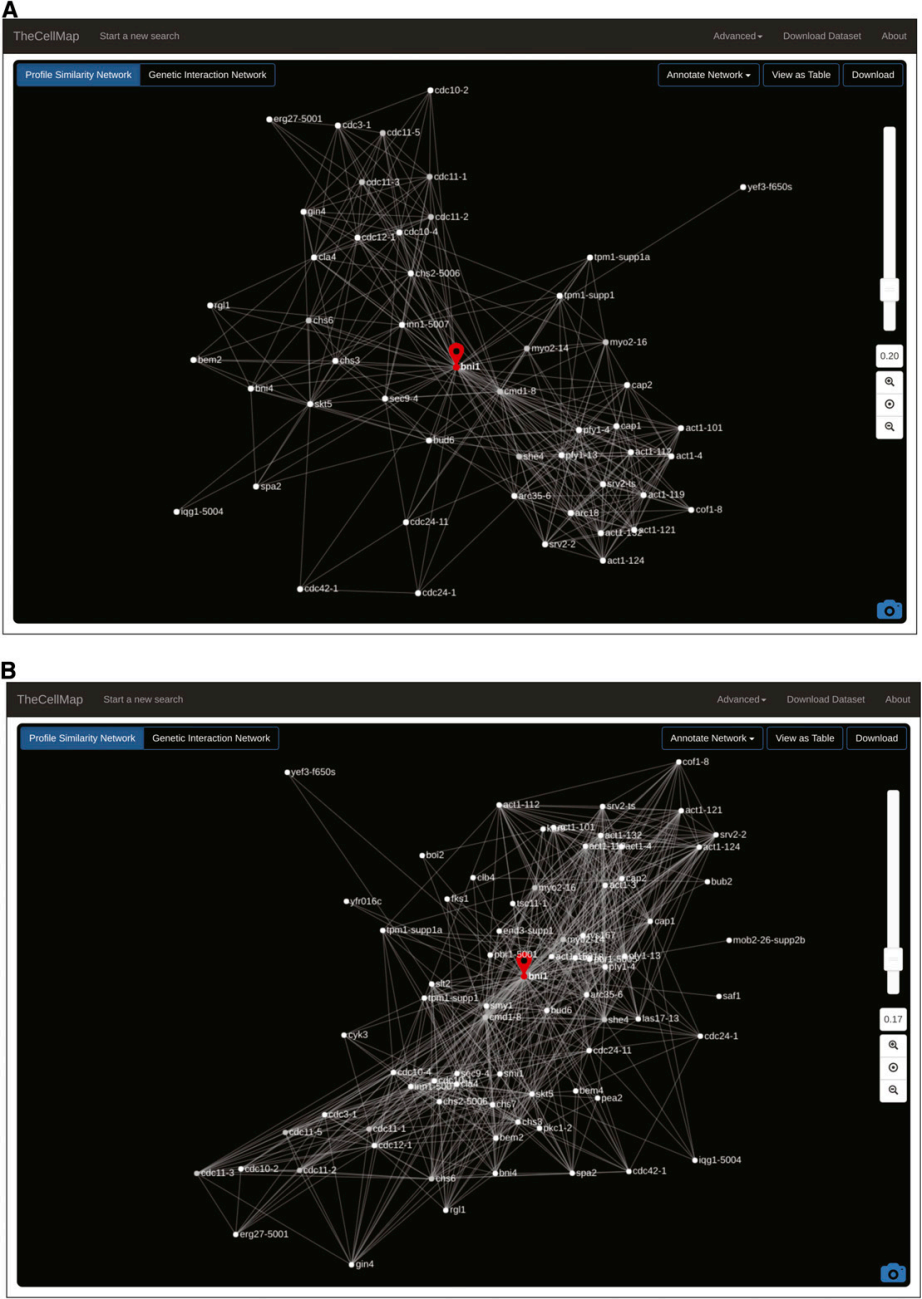
Genetic interaction profile similarity subnetworks. (A) A *BNI1*-specific profile similarity subnetwork with standard profile similarity threshold (PCC > 0.2) applied. Genes that have similar genetic interaction profiles to *BNI1* are shown and positioned in an unbiased manner based on the extent of their similarity to the *BNI1* profile. (B) A *BNI1*-specific profile similarity subnetwork with a more lenient profile similarity threshold (PCC > 0.17) applied. PCC, Pearson correlation coefficient.

Once a gene’s location is determined, the connections that define its position on the global network of genetic interaction profiles can be visualized by extracting a subnetwork for the gene(s) of interest. The “Extract Subnetwork” feature selects the genes that are directly connected to, and thus share similar profiles to, the gene of interest, which means they have many genetic interactions in common (Figure 2). The same edge-weighted, spring-embedded layout algorithm is applied to reposition nodes using only the connections present in the subnetwork (Figure 4). Importantly, the strict threshold on profile similarity applied to the global network is no longer imposed on smaller subnetworks, although the default setting for initial production of the subnetwork is the same as for the global network (PCC = 0.20) (Figure 4A). At this level of resolution, a user can define the similarity threshold, above which a connection to the gene of interest will appear on the network, by using the slide bar to manually adjust the PCC threshold. After adjusting the similarity threshold, the same layout algorithm automatically reorganizes the network based on the presence of new nodes when the similarity threshold is decreased (Figure 4B) or the absence of nodes lost by increasing the similarity threshold. Note that node position can be changed manually by clicking on and dragging specific nodes and individual nodes can also be manually deleted by selecting the “Delete Node” option from the right click menu.

**Figure 5:**
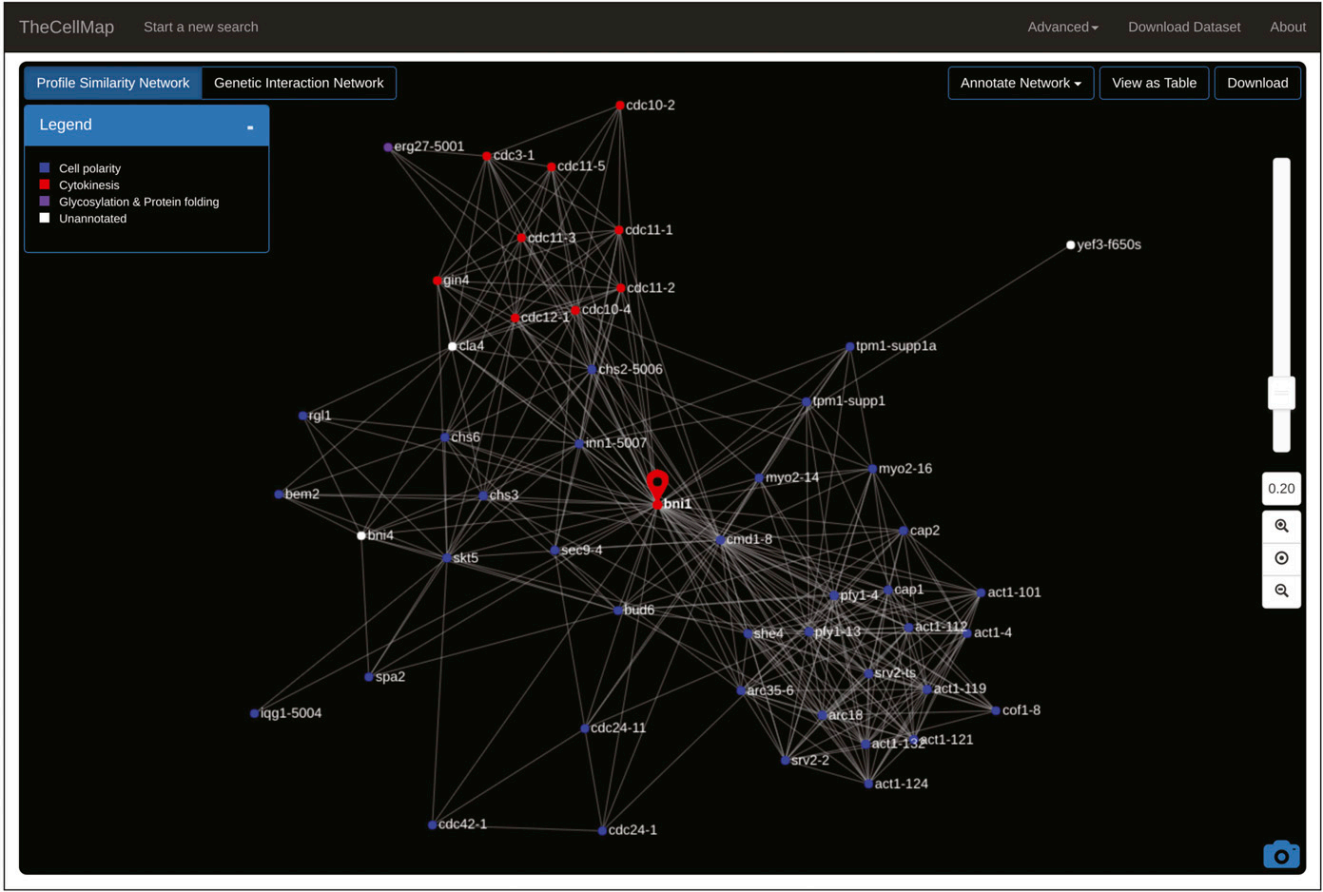
Annotating genetic interaction profile similarity networks. Nodes in the *BNI1* subnetwork are annotated using the SAFE (Spatial Analysis of Functional Enrichment) function standard available from the “Annotate Network” dropdown menu. The sliding bar to the right allows the user to adjust the stringency requirement for adding genes to the network (see text for details).

A subnetwork can also be annotated using different functional standards available from the “Annotate Network” dropdown menu, including the SAFE functional standard (Figure 5). As previously described (Baryshnikova 2016), the SAFE method identifies dense network regions associated with specific functional attributes. Applying SAFE to the global genetic interaction profile similarity network identified 569 significantly enriched Gene Ontology (GO) bioprocess terms (Ashburner *et al.* 2000) that mapped to 19 unique network regions and covered 1480 genes. The SAFE functional standard is composed of 19 general terms that summarize the GO bioprocesses that map to each network region (Costanzo *et al.* 2016). After selecting a functional standard, nodes annotated to the same functional term will be colored similarly (Figure 5). For example, a *BNI1* genetic interaction profile similarity subnetwork annotated using the SAFE functional standard highlighted distinct clusters corresponding to functions that depend on *BNI1*-mediated actin nucleation (Figure 5) (Evangelista *et al.* 2003). Other available functional standards include a custom set of biological process terms (Costanzo *et al.* 2010), GO Slim (Harris *et al.* 2004), a protein complex standard (Costanzo *et al.* 2016), and two different protein localization standards (Huh *et al.* 2003; Koh *et al.* 2015). A user can also upload a custom functional standard from the “Annotate Network” menu as well as change node, and edge attributes from the “Advanced” settings menu.

### Dubious genes and neighboring gene effects

The yeast deletion mutant collection used in our genetic interaction studies contains strains deleted for ORFs (open reading frames) annotated as “dubious,” which are unlikely to encode an expressed protein because the ORF is not conserved in other *Saccharomyces* species and/or there is no experimental evidence that a gene product is produced from the ORF (http://www.yeastgenome.org/help/general-help/glossary). Dubious ORFs tend to be located in very close proximity and often overlap the coding sequence of a neighboring ORF. As a result, deletion of a dubious gene can result in partial deletion of a characterized neighboring gene. Thus, the genetic interaction profile associated with a dubious gene often resembles the profile of the neighboring gene. We chose to include genetic interaction profiles of dubious genes in the genetic profile similarity network, represented as open circles on the networks, because these profiles can serve as controls for screen quality or as a proxy for the genetic interaction profile of the neighboring, verified gene.

**Figure 6:**
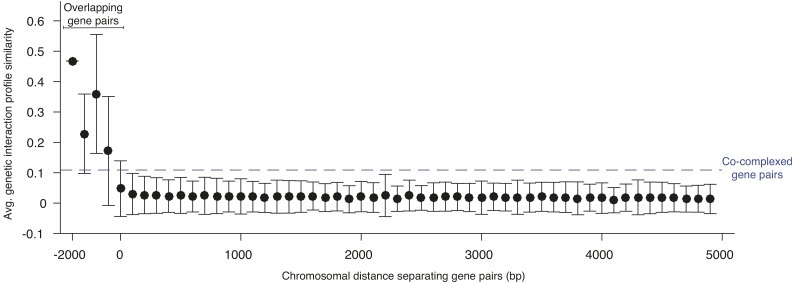
Neighbor gene effects. Graph showing the average genetic interaction profile similarity and SD of gene pairs located on the same chromosome (y axis) and separated by a defined distance (x axis). Negative values indicate gene pairs with physically overlapping coding sequences. The average genetic interaction profile similarity of gene pairs annotated to the same protein complex (*i.e.*, cocomplexed gene pairs) is indicated (dashed blue line).

Similar to the effects of deleting dubious genes, the phenotype observed in a deletion mutant strain can sometimes reflect the combined biological consequence of inactivating both the gene of interest and the adjacent ORF. Thus, it is possible that the interactions comprising the genetic profile of a gene may, in some cases, reflect both its own interactions as well as the interactions of its neighbor (Ben-Shitrit *et al.* 2012). A recent study explored the extent of neighboring gene effects in a previous version of the genetic interaction network and suggested that a fraction of negative genetic interactions could be explained by this effect (Atias *et al.* 2016). To explore the neighbor gene effect in the global genetic network, we examined the relationship between genetic interaction profile similarity and the physical distance separating pairs of genes on the same chromosome (Figure 6). If mutation of a particular gene also compromises the function of an adjacent gene, then the two genes should share many genetic interactions in common and exhibit similar genetic interaction profiles. Unsurprisingly, ORFs whose coding sequence physically overlapped one another exhibited strong profile similarity (Figure 6). However, nonoverlapping gene pairs separated by as little as 200 bp shared significantly less profile similarity than the average pair of genes annotated to the same protein complex (Figure 6). Thus, while individual genetic interactions can be affected by neighboring gene effects (Atias *et al.* 2016), our analysis suggests that the strongest genetic interactions, which drive profile similarity, are mostly unaffected by disruption of a neighboring gene. Based on our analysis, we estimate that only a small fraction of gene pairs exhibit a neighboring gene effect with the potential to impact profile similarity. Applying relatively conservative criteria (neighboring gene pairs with a genetic interaction profile similarity of PCC > 0.1 and separated by <400 bp), we identified 392 genes (<7% of all genes tested) with genetic interaction profiles that may be susceptible to a potential neighboring gene effect. These genes are represented as gray nodes in the profile similarity network (Supplemental Material, Table S1).

### Exploring negative and positive genetic interaction networks

**Figure 7:**
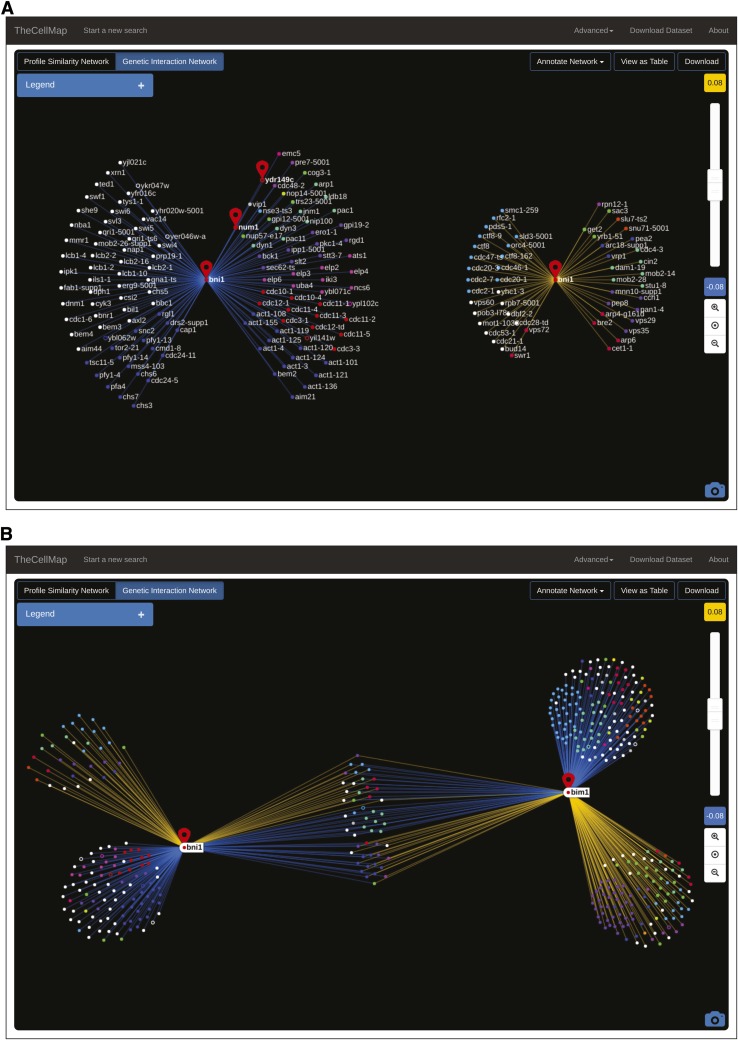
Negative and positive genetic interaction subnetworks. (A) A genetic interaction network illustrating negative (blue) and positive (yellow) genetic interactions for *BNI1. NUM1* and *YDR149C* are also indicated. Nodes are colored based on the SAFE annotation standard. Open circles represent dubious ORFs. (B) A *BNI1* and *BIM1* genetic interaction network. Negative and positive interactions are depicted as in (A). Nodes are colored based on the SAFE annotation standard. Open circles represent dubious ORFs. ORF, open reading frame; SAFE, Spatial Analysis of Functional Enrichment.

Due to the large number of interactions, a global network of negative and positive interactions is complex and difficult to visualize. However, visualization of direct negative and positive genetic interactions is possible after extracting a genetic profile similarity subnetwork for an individual gene or small subsets of genes. Transitioning from profile similarity to direct genetic interaction networks is achieved by selecting the “Genetic Interaction Network” button (Figure 4). A user can switch back to the profile similarity subnetwork at any time by selecting the “Profile Similarity Network” button (Figure 4). In the case of a single gene (*e.g.*, *BNI1* specific network; Figure 7A), two separate genetic interaction networks are generated, one illustrating negative interactions and a second showing positive interactions. Genetic interaction partners are organized in a circular arrangement and are connected to the gene of interest by negative (blue) or positive (yellow) edges (Figure 7A). As a default, only those negative and positive interactions that satisfy a previously defined confidence threshold (*P*-value < 0.05 and |SGA score| > 0.08) (Costanzo *et al.* 2010, 2016) are plotted on the network. The default confidence threshold was determined based on several evaluation metrics including reproducibility of genetic interactions measured from independent replicate experiments, reproducibility of interactions identified among available reciprocal gene pairs (*i.e.*, query A-array B *vs.* query B-array A), and the extent to which enrichment for GO coannotated gene pairs was correlated with the significance and magnitude of genetic interaction scores (Baryshnikova *et al.* 2010; Costanzo *et al.* 2010, 2016). As with the profile similarity thresholds (Figure 4), a slide bar enables manual adjustment of thresholds applied to the negative and positive genetic interaction scores (Figure 7). Nodes will disappear when more stringent interaction score thresholds are set whereas additional nodes will appear in the network at more lenient score thresholds. Node position in an unannotated network is determined by genetic interaction strength. Genes with more extreme interactions are positioned closer to the gene of interest near the center of the network, while genes with weaker interactions are located further away and closer to the network periphery (Figure 7A). Negative and positive genetic interaction networks can also be annotated using the same functional standards described above, and node position within the networks will be reorganized in an annotation-dependent manner such that genes annotated to the same functional term will be grouped together (Figure 7A). As with the profile similarity network, open circles represent dubious ORFs. For example, *BNI1* shows a negative interaction with the dubious ORF *YDR149C*, which partially overlaps its neighboring gene *NUM1*. Thus, the observed negative interaction with *BNI1* likely reflects the fact that deletion of *YDR149C* also disrupts the function of the *NUM1* gene, which also shows a negative interaction with *BNI1* (Figure 7A).

Unlike the “spoke” layout used to visualize genetic interactions for a single gene (Figure 7A), selecting multiple genes requires a different network layout (Figure 7B). For example, when two genes are selected, negative and positive interactions unique to each gene of interest are positioned on the periphery of the network whereas shared interactions are located between the selected genes and grouped according to interaction type, such that negative and positive interactions are clearly separated (Figure 7B). Node position and attributes, such as color and size, can also be changed manually as described for profile similarity subnetworks (Figure 4).

### Generating and downloading gene-specific interaction lists

**Figure 8:**
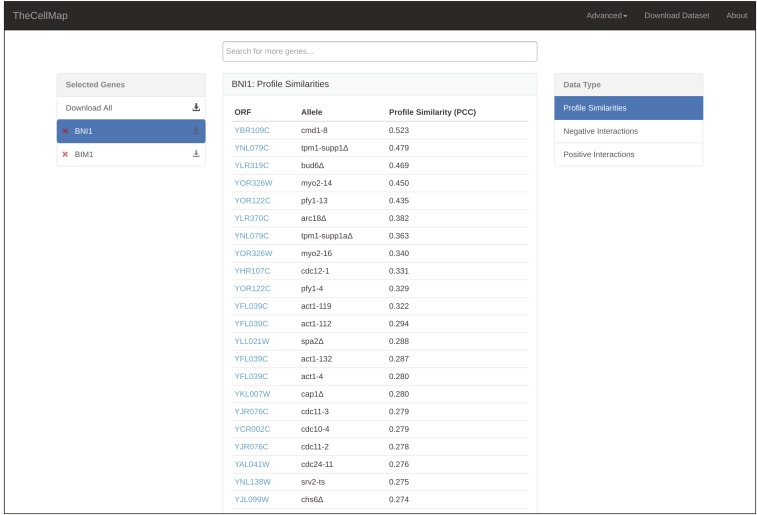
Gene-specific genetic interaction data. A screenshot illustrating genetic interaction data organized in a tabular format. ORF, open reading frame; PCC, Pearson correlation coefficient.

In addition to visualizing the yeast genetic interaction dataset in the form of profile similarity or direct genetic interaction networks, data for a specific gene or subset of genes can also be viewed in a tabular format (Figure 8). The table format can be accessed by clicking the “View as Table” button available from the global network page (Figure 2A), the subnetwork profile similarity pages (Figure 4), or the genetic interaction network page (Figure 7). The table view contains two menus: “Selected Genes” and “Data Types” (Figure 8). The Selected Genes menu lists the genes selected as part of the search and the list expands as more genes are added to a search. Only data corresponding to the highlighted gene in the Selected Genes menu will be shown in the table. The second menu indicates the type of data, either profile similarities, negative genetic interactions, or positive genetic interactions, which is shown in the table for a highlighted gene (Figure 8). As a default, profile similarities and interactions that satisfy standard confidence thresholds, described above, are listed in the table. However, a complete list of interactions can be viewed by selecting the “Load All” button at the bottom of the table. Finally, data for any gene of interest can be downloaded directly from the Table View page (Figure 8) or by selecting the “Download” button available from the global network page (Figure 2A), subnetwork profile similarity pages (Figure 4), or the genetic interaction network page (Figure 7). Data for a selected gene(s) will be downloaded as a Microsoft Excel (.xls) file and includes all three data types: profile similarities and negative and positive interactions corresponding to the gene(s) of interest.

### Conclusions

A global genetic interaction network has been mapped for a model eukaryotic cell, the budding yeast *S. cerevisiae*. TheCellMap.org provides a single platform to facilitate access, visualization, and analysis of the global yeast genetic interaction network. Note that we continue to map genetic interactions for remaining gene pairs and new data will be deposited in the database as it is generated. We also continue to develop new functionality, including tools to integrate profile similarities and direct genetic interactions into a single network, similar to networks shown in our previous study [see Figure 9 in Costanzo *et al.* (2016)]. In addition, we will also expand TheCellMap.org to allow users the ability to upload and integrate their own phenotypic data with the genetic interaction network as previously shown with chemical genetic interaction data (Costanzo *et al.* 2010). We anticipate that the tools developed as part of this web-accessible database will provide valuable insights into phenotypic and functional connections between different genes, as well as a powerful resource for gene function discovery and the analysis of the general principles of genetic interaction networks.

## Supplementary Material

Supplemental material is available online at www.g3journal.org/lookup/suppl/doi:10.1534/g3.117.040220/-/DC1.
